# *DNAH6* and Its Interactions with PCD Genes in Heterotaxy and Primary Ciliary Dyskinesia

**DOI:** 10.1371/journal.pgen.1005821

**Published:** 2016-02-26

**Authors:** You Li, Hisato Yagi, Ezenwa Obi Onuoha, Rama Rao Damerla, Richard Francis, Yoshiyuki Furutani, Muhammad Tariq, Stephen M. King, Gregory Hendricks, Cheng Cui, Manush Saydmohammed, Dong Min Lee, Maliha Zahid, Iman Sami, Linda Leatherbury, Gregory J. Pazour, Stephanie M. Ware, Toshio Nakanishi, Elizabeth Goldmuntz, Michael Tsang, Cecilia W. Lo

**Affiliations:** 1 Department of Developmental Biology, University of Pittsburgh School of Medicine, Pittsburgh, Pennsylvania, United States of America; 2 Division of Pediatric Cardiology, Tokyo Women’s Medical University, Tokyo, Japan; 3 Departments of Pediatrics and Medical and Molecular Genetics, Indiana University School of Medicine, Indianapolis, Indiana, United States of America; 4 University of Connecticut Health Center, Farmington, Connecticut, United States of America; 5 The Core Electron Microscopy Facility, University of Massachusetts Medical School, Worcester, Massachusetts, United States of America; 6 Department of Pulmonary and Sleep Medicine, Children’s National Medical Center, Washington, D.C., United States of America; 7 Department of Cardiology, Children’s National Medical Center, Washington, D.C., United States of America; 8 Program in Molecular Medicine, University of Massachusetts Medical School, Worcester, Massachusetts, United States of America; 9 Department of Pediatrics, University of Pennsylvania Perelman School of Medicine, Division of Cardiology, Children’s Hospital of Philadelphia, Philadelphia, Pennsylvania, United States of America; Washington University School of Medicine, UNITED STATES

## Abstract

Heterotaxy, a birth defect involving left-right patterning defects, and primary ciliary dyskinesia (PCD), a sinopulmonary disease with dyskinetic/immotile cilia in the airway are seemingly disparate diseases. However, they have an overlapping genetic etiology involving mutations in cilia genes, a reflection of the common requirement for motile cilia in left-right patterning and airway clearance. While PCD is a monogenic recessive disorder, heterotaxy has a more complex, largely non-monogenic etiology. In this study, we show mutations in the novel dynein gene *DNAH6* can cause heterotaxy and ciliary dysfunction similar to PCD. We provide the first evidence that trans-heterozygous interactions between *DNAH6* and other PCD genes potentially can cause heterotaxy. *DNAH6* was initially identified as a candidate heterotaxy/PCD gene by filtering exome-sequencing data from 25 heterotaxy patients stratified by whether they have airway motile cilia defects. *dnah6* morpholino knockdown in zebrafish disrupted motile cilia in Kupffer’s vesicle required for left-right patterning and caused heterotaxy with abnormal cardiac/gut looping. Similarly *DNAH6* shRNA knockdown disrupted motile cilia in human and mouse respiratory epithelia. Notably a heterotaxy patient harboring heterozygous *DNAH6* mutation was identified to also carry a rare heterozygous PCD-causing *DNAI1* mutation, suggesting a *DNAH6/DNAI1* trans-heterozygous interaction. Furthermore, sequencing of 149 additional heterotaxy patients showed 5 of 6 patients with heterozygous *DNAH6* mutations also had heterozygous mutations in *DNAH5* or other PCD genes. We functionally assayed for *DNAH6/DNAH5* and *DNAH6/DNAI1* trans-heterozygous interactions using subthreshold double-morpholino knockdown in zebrafish and showed this caused heterotaxy. Similarly, subthreshold siRNA knockdown of *Dnah6* in heterozygous *Dnah5* or *Dnai1* mutant mouse respiratory epithelia disrupted motile cilia function. Together, these findings support an oligogenic disease model with broad relevance for further interrogating the genetic etiology of human ciliopathies.

## Author Summary

Heterotaxy is a birth defect involving randomization of left-right body axis. Its genetic etiology is still poorly understood, but recent studies suggest mutations in genes causing primary ciliary dyskinesia (PCD), a sinopulmonary disease, also can cause heterotaxy. Moreover, heterotaxy patients can show airway cilia dysfunction reminiscent of PCD. The link between these two seemingly disparate diseases reflects the common requirement for motile cilia in both left-right patterning and airway mucus clearance. Sequencing analysis of heterotaxy patients together with experimental modeling identified *DNAH6* as a novel gene that can cause both heterotaxy and PCD. We further showed *DNAH6* can interact with other PCD genes to mediate a more complex oligogenic etiology of disease. Thus experimental modeling with double gene knockdown showed digenic interactions of *DNAH6* with *DNAH5* or *DNAI1* could disrupt motile cilia function in the respiratory epithelia and also cause heterotaxy in zebrafish embryos. These findings provide the first experimental evidence indicating oligogenic interactions can contribute to the complex genetics of heterotaxy.

## Introduction

Heterotaxy and primary ciliary dyskinesia (PCD, OMIM: 24440) are both rare heritable disorders with prevalence of approximately 1 in 10,000[1, 2]. While heterotaxy patients exhibit defects in patterning of the left-right body axis, PCD patients suffer sinopulmonary disease due to airway mucus clearance defects caused by immotile or dyskinetic respiratory cilia in the airway. These two seemingly disparate diseases may have overlapping genetic etiology, as PCD patients also can exhibit laterality defects, comprising either of complete reversal of visceral organ situs (situs inversus) as in Kartagener’s syndrome or randomization of visceral organ situs as in heterotaxy. This likely reflects the common requirement for motile cilia in the embryonic node for left-right patterning and in the airway for mucociliary clearance.

A link between PCD and heterotaxy is further supported by the recent finding of a high prevalence of airway ciliary dysfunction similar to that seen with PCD in heterotaxy patients with congenital heart disease (CHD). This is of clinical importance, as heterotaxy is highly associated with complex CHD and CHD/heterotaxy patients are known to have high postsurgical morbidity and mortality associated with more respiratory complications[3]. These findings suggest the poor outcome in these patients may be impacted by mucociliary clearance defects in the airway. Consistent with this, a prospective study of CHD/heterotaxy patients showed those with increased airway ciliary dysfunction exhibited more respiratory symptoms and disease, and worse postsurgical outcomes[4, 5]. Hence, insights into the genetic overlap between heterotaxy and PCD may have relevance for clinical management of this highly vulnerable CHD patient population.

PCD is a monogenic recessive disorder that is genetically heterogeneous with over 30 PCD genes identified. These mostly encode proteins in cilia or are required for motile cilia function. As these genes only account for approximately 60% of PCD cases[6], there are likely additional PCD genes to be identified. A role for PCD genes in heterotaxy is suggested not only by the requirement for motile cilia function in left-right patterning, but also the finding that heterotaxy patients have a high prevalence of airway ciliary dysfunction (CD) similar to that seen with PCD[5]. While heterotaxy has been shown to be highly heritable, its genetic etiology is more complex and is largely non-monogenic in etiology [7, 8]. To date, mutations in over 15 left-right patterning genes have been clinically implicated in heterotaxy, but this accounts for less than 20% of the heterotaxy cases[9–11]. We previously observed heterotaxy patients with airway ciliary dysfunction are enriched for mutations in PCD genes[5], but interestingly these were all heterozygous, including a loss-of-function *DNAI1* mutation known to cause PCD. This would suggest a more complex genetic model of disease mediated by heterozygous mutations in multiple PCD/cilia related genes, ie. trans-heterozygous interactions. Such a model of disease is attractive, given ciliogenesis is a complex multi-step biological process involving hundreds of proteins and mediated by many large multiprotein complexes. As yet, such multigenic interactions in heterotaxy have not been experimentally investigated.

In the present study, we functionally tested the potential contribution of an oligogenic model of disease in heterotaxy, focusing our analysis on PCD/cilia-related genes. Exome sequencing analysis of heterotaxy patients identified dynein gene *DNAH6* as a new heterotaxy/PCD candidate gene. We showed *DNAH6* knockdown disrupted motile cilia function in the human and mouse airway, and in zebrafish Kupffer’s vesicle. Furthermore, *Dnah6* knockdown caused heterotaxy in zebrafish embryos. We further showed dual haploinsufficiency of *Dnah6* and either *Dnai1* or *Dnah5* to model trans-heterozygous *Dnah6/Dnai1 and Dnah6/Dnah5* interactions can cause airway ciliary dysfunction and heterotaxy. These findings show *DNAH6* is required for motile cilia function mediating airway clearance and left/right patterning, and this may involve oligogenic interactions that can contribute to the complex genetics of heterotaxy and PCD.

## Results

### *DNAH6* mutations in heterotaxy patients with ciliary dysfunction

**Fig 1:**
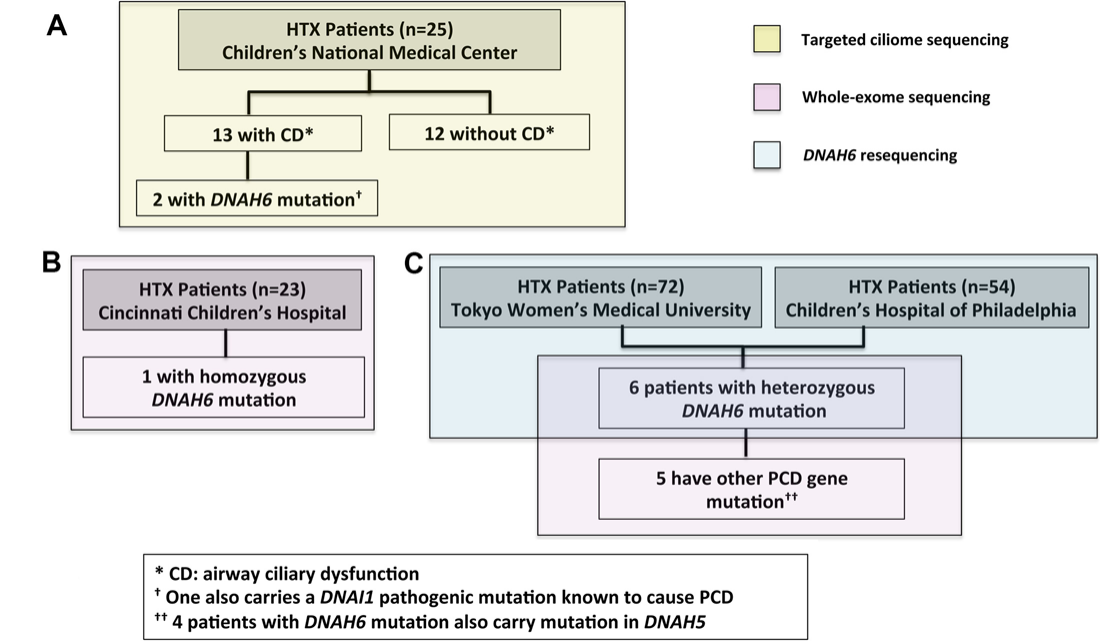
Heterotaxy patient cohorts analyzed by targeted and whole exome sequencing analyses. **(A)** 25 heterotaxy (HTX) patients from Children’s National Medical Center were sequence analyzed with targeted ciliome sequencing (yellow) to identify cilia-related mutations. This includes 13 HTX patients with airway ciliary dysfunction (CD) and 12 patients with normal airway cilia function (without CD). This analysis identified *DNAH6* as the only candidate gene with mutations found exclusively in patients with CD (n = 2). **(B)** Whole-exome sequencing (pink) was conducted in 23 HTX patients from Cincinnati Children’s Hospital, with 1 patient identified with a novel homozygous *DNAH6* mutation. **(C)** *DNAH6* amplicon resequencing (blue) was conducted on 72 HTX patients from Tokyo Women’s Medical University and 54 patients from Children’s Hospital of Philadelphia. Of these 126 patients, 6 were found to have heterozygous *DNAH6* mutation. The latter 6 patients were further analyzed by whole exome sequencing (pink). In 5 of these patients, additional heterozygous mutations were found in other PCD genes including, 4 mutations in *DNAH5*.

**Fig 2:**
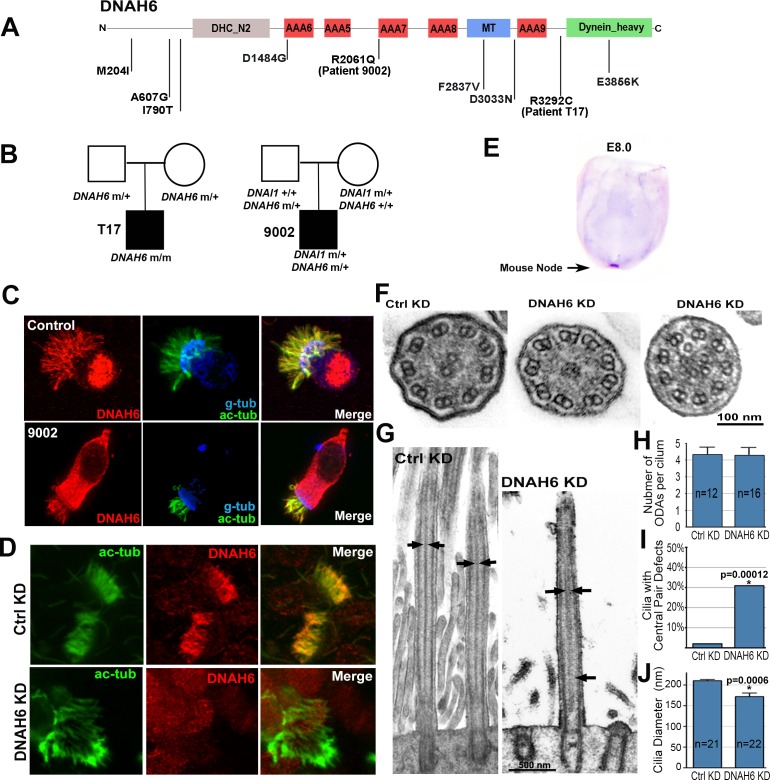
*DNAH6* mutations and the role of *DNAH6* in motile cilia function. **(A)** Schematic of DNAH6 protein structure, predicted functional domains are shown together with the position of novel and rare mutations identified in heterotaxy patients. **(B)** Pedigrees show heritable transmission of homozygous *DNAH6* mutation in patient T17 and double heterozygous *DNAI1*/*DNAH6* mutations in heterotaxy patient 9002. **(C)** DNAH6 antibody (red) is localized in the ciliary axoneme (green) in control human respiratory epithelium and in patient 9002. **(D)** DNAH6 (red) antibody staining is lost in the ciliary axoneme (green) with sh*DNAH6* knockdown of human respiratory epithelia. **(E)** Whole-mount in situ hybridization analysis showed *Dnah6* is exclusively expressed in the embryonic node of E8.0 mouse embryo. **(F-J)** Electron microscopy of human airway epithelia after *DNAH6* knockdown showed missing central pair and extra microtubules vs. 9+2 cilia ultrastructure in normal airway cilia (F). This also can be observed in longitudinal views (arrows in G). Quantitation using EM cross sections showed no ODA defects (H), confirmed the central pair defects (I, Chi-square test, p-value = 1.2x10^-4^)**,** and also showed reduction in cilia diameter (J, two-tailed Student t-test, p-value = 0.0006).

To investigate the role of cilia and PCD mutations in CHD/heterotaxy, we carried out targeted exome sequencing analysis of 25 CHD/heterotaxy patients from Children’s National Medical Center who were previously assessed for motile cilia function in the airway– 13 were shown to have defects in motile cilia function, referred to as having ciliary dysfunction (CD) and 12 exhibit normal motile cilia function, referred to as having no-CD [5]. The sequencing analysis was focused on ~900 cilia related or ciliome genes (S1 Table), interrogating for novel or rare coding variants present only in heterotaxy patients with CD, but not in those without CD (<0.8% allele frequency; see Materials and Methods). This analysis recovered *DNAH6* as the only ciliome candidate gene, with two heterotaxy patients with CD identified with heterozygous *DNAH6* mutations (Fig 1A). *DNAH6* is predicted to be an inner dynein arm component based on evolutionary conservation with cilia genes in *Chlamydomonas*. It is an ortholog of *Chlamydomonas* DHC2, an inner dynein arm component essential for motile cilia function. Interestingly, one of the two patients identified with a *DNAH6* mutation is 9002, a patient previously found to have a heterozygous *DNAI1* PCD founder mutation. Genotyping analysis showed the *DNAH6* mutation in patient 9002 was inherited from the unaffected father, while the pathogenic *DNAI1* mutation was inherited from the unaffected mother (Fig 2B and S1 Fig).

### *DNAH6* required for airway ciliary motility

The role of DNAH6 in motile cilia function has not been investigated previously, but DNAH6 antibody staining showed DNAH6 is abundantly expressed in the human airway ciliary axoneme (Fig 2C). *Dnah6* transcripts are also highly expressed in the mouse embryonic node where motile cilia play an important role in establishing the left-right axis (Fig 2E). To assess the role of *DNAH6* in cilia motility, we developed an assay to assess gene function required for ciliogenesis and cilia function in the airway. This entailed generating primary explants of human nasal biopsies or mouse tracheal epithelia for ex vivo analysis. The explanted tissue is first cultured under conditions that favor deciliation and expansion with proliferative growth as an adherent monolayer. This is followed by change in culture conditions that promote reciliation with growth in suspension culture. With this cycle of deciliation/reciliation, lentiviral shRNA mediated gene knockdown can be used to assay gene function required for ciliogenesis and motile cilia function. Lentiviral mediated *DNAH6* shRNA gene knockdown in this ex vivo respiratory epithelia culture resulted in the near complete ablation of *DNAH6* transcript and protein expression (Fig 2D and S2 Fig). This resulted in very sparse ciliation in the reciliating respiratory epithelia, and the few cilia found were short (Fig 2D) and mostly immotile, with some exhibiting occasional stiff dyskinetic ciliary motion (S2 Movie). This contrasts with the high ciliation density (Fig 2D) and normal rapid synchronous ciliary beat seen in control cultures treated with scrambled shRNA (S1 Movie). We note previous nasal biopsy of patient 9002 showed abnormally short and sparse cilia, and videomicroscopy showed the cilia were mostly immotile. Here we further show with immunostaining, that DNAH6 was retained in the ciliary axoneme of patient 9002 (Fig 2C). This is not unexpected, since the patient has a heterozygous wildtype *DNAH6* allele.

We also examined the effects of *DNAH6* knockdown on cilia ultrastructure in the respiratory epithelia, as cilia ultrastructural defects are often observed with PCD[12], Analysis of cilia ultrastructure by electron microscopy (EM) showed the abundance of outer dynein arm (ODA) was not affected by *DNAH6* knockdown (Fig 2F and 2H). This was confirmed by the finding of robust DNAH5 immunostaining in the ciliary axoneme in the reciliated airway cultures subject to *DNAH6* knockdown (S3 Fig). Assessment of the inner dynein arm component (IDA) by immunostaining with an antibody to the IDA component DNALI1 also showed robust DNALI localization in the ciliary axoneme in respiratory epithelia subjected to *DNAH6* knockdown (S3 Fig). Together these findings show ODA and IDA assembly were not affected by *DNAH6* deficiency. However, EM ultrastructural analysis showed an unexpected cilia central pair defect with *DNAH6* knockdown. This can be observed in both the cross section (Fig 2F) and longitudinal (Fig 2G) views. In cross section views, there may be absent central pair or multiple randomly placed accessory microtubules in place of the central pair (Fig 2F). Quantitation using the cross-section EM images showed after *DNAH6* knockdown, 30% of the respiratory cilia have central pair defects (p = 1.2x10^-4^, Fig 2I). Immunostaining analysis with an antibody to central pair component RSPH4A did not show any change in RSPH4A expression or localization with *DNAH6* knockdown (S3 Fig). The EM analysis also showed a reduction in the caliper of the cilia with *Dnah6* knockdown (p = 0.0006, Fig 2J). The ciliation defects led us to further examine whether the expression of ciliogenesis regulators *CCNO*, *MCIDAS* and *FOXJ1* were altered. Analysis of the mouse and human respiratory epithelia showed the expression of these three genes were not altered with *DNAH6* knockdown (S4 Fig). Consistent with these results, TEM analysis of the reciliating respiratory epithelia showed no basal body docking defects. Similarly, *dnah6* MO knockdown in zebrafish embryos did not alter the robust expression of *foxj1* in Kupffer’s vesicle (S4 Fig). Together these findings show *DNAH6* deficiency causes ciliogenesis defect associated with the disruption of cilia ultrastructure, but not with the activation of the ciliogenesis program.

### *DNAH6* required for motile cilia function mediating left-right patterning

**Fig 3:**
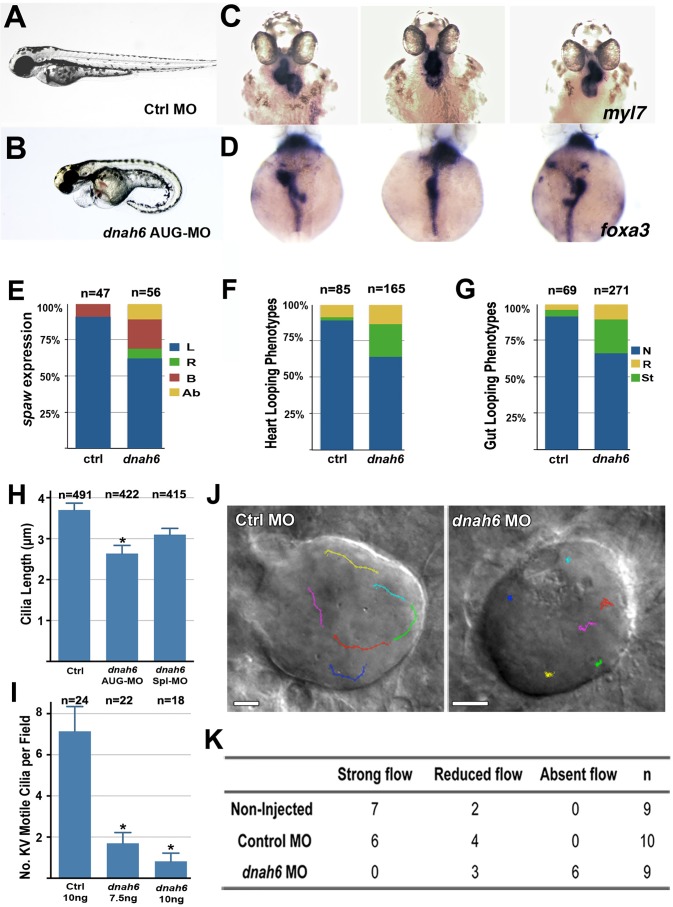
*dnah6* morpholino knockdown in zebrafish embryo cause laterality defects. **(A,B)** *dnah6* MO injected embryos exhibited curly tail and cardiac edema phenotype at 48 hours post fertilization (hpf). **(C,D)** *dnah6* MO injected embryos at 48hpf exhibited heart (C) and gut (D) looping defects, including failure to loop (middle panels) and reversal looping (right panels). **(E)** In *situ* hybridization analysis revealed abnormal right sided (R), bilateral (B), and absent (Ab) *spaw* expression after *dnah6* MO knockdown. **(F,G)** Heart/gut looping defects were observed in *dnah6 MO*-injected embryos, including normal (N), right sided (R), and straight (St) heart/gut looping phenotypes. **(H).** KV cilia were shorter (middle) in *dnah6* AUG-MO but not *dnah6* spl-MO injected embryos (p-value = 0.0017). **(I)**. High-speed videomicroscopy showed reduction in KV motile cilia of *dnah6* MO injected embryos (p = 1.6x10^-4^ for 7.5 ng and p = 1.1x10^-5^ for 10 ng *dnah6* MO; two-tailed Student’s t-test). **(J)** Little bead movement (color tracing) was observed in *dnah6* MO injected embryos. **(K)** KV flow was absent or reduced with *dnah6* knockdown (Chi-square test, p-value = 0.0192).

To investigate the role of *DNAH6* in left-right patterning, we conducted *dnah6* antisense morpholino (MO) knockdown analysis in zebrafish embryos. We observed *dnah6* MO knockdown caused a constellation of phenotypes associated with heterotaxy. This included abnormal curved body axis (Fig 3A and 3B), perturbation in the orientation of heart and gut looping (Fig 3C, 3D, 3F and 3G) and disruption of left-sided *spaw* expression (zebrafish *Nodal* homolog) (Fig 3E). Similar left right patterning defects were elicited by both the translation blocking (AUG-MO) and splice-blocking MO (Spl-MO), with the latter yielding lower efficiency (S5 Fig). We demonstrated this is likely due to the presence of maternal *dnah6* transcripts (S6 Fig and S7 Fig).

Analysis of Kupffer’s vesicle (KV), the zebrafish embryonic structure with motile cilia that is equivalent to the mouse embryonic node, revealed no change in the total number of cilia (S8 Fig), but rather a marked reduction in KV cilia length and decrease in the number of motile KV cilia (Fig 3H). KV ciliary motion was highly dyskinetic (S3 Movie), with little or no effective flow (Fig 3J and S4 Movie) as compared to control (Fig 3K; p-value = 0.0293, Chi-square test). These findings confirm *dnah6* is required for motile cilia function and the specification of the left-right body axis in the zebrafish embryo. We note it was not possible to directly test for MO rescue of the various human *DNAH6* mutations, given the very large size of the *DNAH6* coding sequence precluded construction of a *dnah6* expression construct. Nevertheless, together these ex vivo and in vivo gene knockdown analyses demonstrated *DNAH6* is essential for motile cilia function and its deficiency can cause airway ciliary dysfunction and heterotaxy.

### *DNAH6* and PCD mutations in heterotaxy patients

To further assess the potential contribution of *DNAH6* mutations in heterotaxy, we conducted sequencing analysis of an additional 149 heterotaxy patients. This included whole exome sequencing analysis of 23 patients from Cincinnati Children’s Hospital (Fig 1B), and targeted *DNAH6* resequencing of 126 heterotaxy patients from Children’s Hospital of Philadelphia and Tokyo Women’s Medical University (Fig 1C). This combined sequencing analysis identified one patient (T17 from Cincinnati Children’s Hospital) with a novel homozygous *DNAH6* mutation. This mutation is predicted to be pathogenic (Table 1 and S2 Table), and was inherited from unaffected parents who were both heterozygous carriers (Fig 2B). This suggested the possibility that *DNAH6* also can cause heterotaxy in a recessive manner.

**Table 1:** *DNAH6* and PCD gene mutations identified in heterotaxy patients.

Patient	Race/Ethnicity	Sex	Organ Situs	Cilia Function^¥^	Gene	Nucleotide Change	Protein Change	Zygosity	Allele Frequency*
**Children’s National Medical Center**									
9002	White	M	Heterotaxy	*CD*	*DNAI1^†^*	IVS1+2_3insT*^†^*	Truncation*^†^*	het	0.048%
					*DNAH6*	c.6182G>A	p.R2061Q	het	Novel
9027	Hispanic	F	Heterotaxy	*CD*	*DNAH6*	c.4451A>G	p.D1484G	het	Novel
**Cincinnati Children’s Hospital**									
T17	White	M	Heterotaxy	*n*.*d*	*DNAH6*	c.C9874T	p.R3292C	homo	Novel
**Tokyo Women’s Medical University**									
JP2090	Asian	F	Heterotaxy	*n*.*d*.	*DNAH6*	c.G11566A	p.E3856K	het	Novel
					*DNAH5*	c.A737G	p.N246S	het	Novel
JP2637	Asian	F	Heterotaxy	*n*.*d*.	*DNAH6*	c.G612A	p.M204I	het	Novel
JP3617	Asian	F	Heterotaxy	*n*.*d*.	*DNAH6*	c.G9097A	p.D3033N	het	Novel
					*DNAH5*	c.C12472T	p.R4158W	het	0.007%
JP3634	Asian	M	Heterotaxy	*n*.*d*.	*DNAH6*	c.C1820G	p.A607G	het	Novel
					*DNAH5*	c.C12472T	p.R4158W	het	0.007%
**Children’s Hospital of Philadelphia**									
GOLD53	White	F	Heterotaxy	*n*.*d*.	*DNAH6*	c.T2369C	p.I790T	het	0.06%
					*DNAH5*	c.C3514A	p.Q1172K	het	0.2%
					*RSPH4A*	c.C1990T	p.P664S	het	0.001%
GOLD54	Black	M	Heterotaxy	*n*.*d*.	*DNAH6*	c.T8509G	p.F2837V	het	0.01%
					*DNAH11*	c.G13135T	p.G4389C	het	Novel
					*LRRC50*	c.C1582G	p.T531R	het	Novel

^**†**^ known pathogenic PCD causing mutations

^**¥**^ CD: airway ciliary dysfunction as determined by the finding of low nasal nitric oxide and abnormal airway ciliary motion observed by videomicroscopy

* Allele frequencies for rare variants derived from NHLBI exome database

An additional 6 heterotaxy patients were identified with heterozygous *DNAH6* mutations—four entirely novel and two very rare (S2 Table). These 6 patients were further analyzed by whole exome sequencing analysis to determine if there are other ciliome mutations. This recovered 7 additional novel/rare heterozygous coding variants in known PCD genes in 5 of the 6 patients—4 in *DNAH5*, a motor dynein gene commonly associated with PCD (Table 1). Together with patient 9002, 6 of 8 heterotaxy patients with heterozygous *DNAH6* mutations also carried heterozygous mutations in known PCD genes. These findings suggest possible trans-heterozygous interactions between *DNAH6* and *DNAI1*, *DNAH5* or other PCD genes may contribute to an oligogenic model of disease in heterotaxy.

### Assaying *dnah6* interactions with *dnah5* or *dnahi1*

To investigate whether trans-heterozygous interactions between *DNAH6* and other PCD genes can perturb motile cilia function and contribute to heterotaxy and PCD, we examined digenic interactions between *DNAH6* and *DNAI1* or *DNAH5*. This analysis focused on the possible effects of haploinsufficiency using subthreshold morpholino or siRNA gene knockdown, as PCD mutations are often loss of function, such as the splicing defect *DNAI1* founder mutation in patient 9002.

#### Dual haploinsufficiency of *dnah6* with *dnai1* or *dnah5* in heterotaxy

**Fig 4:**
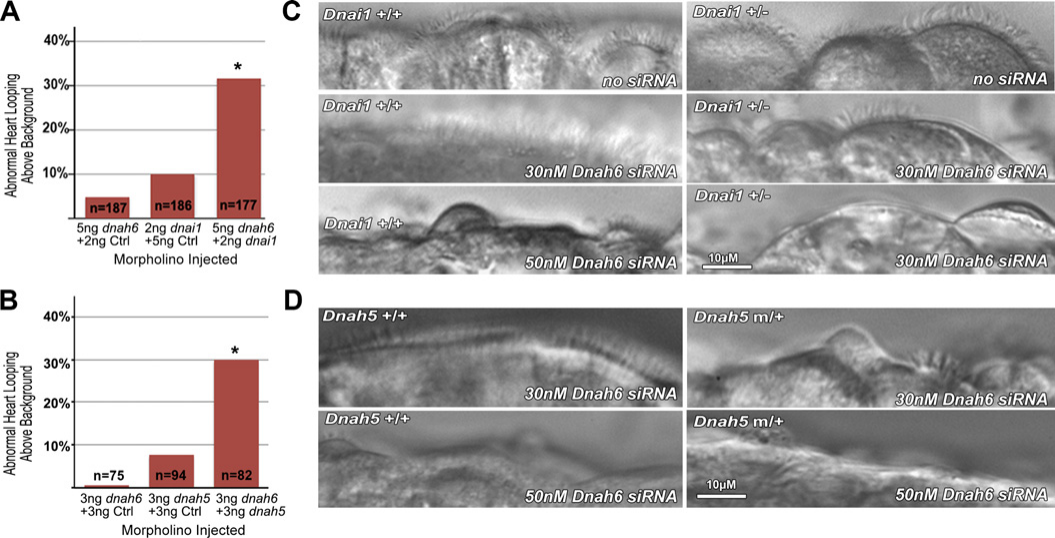
*Dnah6* genetically interacts with *Dnai1* and *Dnah5* to cause heterotaxy and PCD. **(A,B)** Embryos injected with subthreshold dose of *dnah6* and *dnai1* MO show increased heart looping defects compared with Ctrl MO injections (n = 177, p-value = 3.8x10^-8^), or single injection of either *dnai1* (p = 9.29x10^-9^) or *dnah6* (p = 2.04x10^-8^) MO at the same MO dose (A). Similar results were observed with subthreshold *dnah5/dnah6* double MO knockdown (n = 82; p = 1.74x10^-5^, Bonferroni corrected)**. (C,D)** Reciliating mouse airway epithelia from wildtype (+/+) and heterozygous (+/-) *Dnai1* knockout (C) or *Dnah5* mutant (D) mice show robust ciliation and ciliary motion (S5 Movie and S6 Movie). 30nM *Dnah6* siRNA had no effect on ciliation or cilia motility in wildtype airway epithelia, but in heterozygous *Dnai1 or Dnah5* mutant airway, ciliation was reduced and ciliary motion was dyskinetic (S5 Movie and S6 Movie). With 50nM siRNA, little or no cilia was seen in wildtype and heterozygous *Dnai1* or *Dnah5* mutant mouse airway.

To functionally assay for possible digenic interactions in causing heterotaxy, we used subthreshold antisense MO knockdown in zebrafish. Thus we carried out injections of *dnah6/dnai1* or *dnah6/dnah5* MO in zebrafish embryos using subthreshold MO doses that individually had no effect on development. Injections of these subthreshold *dnah6/dnai1* or *dnah6/dnah5* combination resulted in a significant increase in defects of left-right patterning of heart looping (Fig 4A and 4B). In contrast, embryos injected with a control MO or injected singly at the same subthreshold doses of *dnah6*, *dnai1*, or *dnah5* MO showed normal heart looping (Fig 4A and 4B). These findings suggest heterotaxy may arise from trans-heterozygous deficiencies in *dnah6*/*dnai1* or *dnah6/dnah5*.

#### Dual haploinsufficiency of *Dnah6* with *Dnai1* or *Dnah5* in airway ciliary dysfunction

To assess whether digenic trans-heterozygous interactions also may disrupt motile cilia function in the respiratory epithelia similar to that seen with PCD, we took advantage of the availability of *Dnah5* and *Dnai1* mutant mice that are bona fide models of PCD. While heterozygous *Dnah5*^*hlb612*^ or *Dnai1*^*tm1*.*1Leo*^ mutants show normal tracheal cilia motility, homozygous mutants exhibit immotile cilia or cilia with slow/dyskinetic motion [13, 14]. To assay for possible trans-heterozygous interactions between *Dnah6/Dnai1* or *Dnah6/Dnah5*, we used subthreshold siRNA knockdown to model *Dnah6* haploinsufficiency. For these studies, we generated ex vivo primary cultures of the tracheal epithelia from heterozygous *Dnai1* or *Dnah5* mutant mice, and carried out subthreshold *Dnah6* siRNA knockdown.

In untreated reciliating cultures of heterozygous *Dnah5* and *Dnai1* mutant mice, we observed high ciliation density and normal cilia motility (S5 Movie and S6 Movie) indistinguishable from reciliating human airway epithelia or the airway epithelia of wildtype mice. Transfection of the reciliating wildtype mouse tracheal epithelia with subthreshold *Dnah6* siRNA dose (30nM) had no effect on ciliogenesis or cilia motility (Fig 4C and 4D). In contrast, transfection of the same subthreshold siRNA dose (30 nM) in reciliating tracheal epithelia from the heterozygous *Dnai1* or *Dnah5* mutant mice resulted in very sparse ciliation and dyskinetic ciliary motion (Fig 4C and 4D, S5 Movie and S6 Movie), similar to results obtained when higher *Dnah6* siRNA dose (50nM) was used to ablate *Dnah6* function in wildtype respiratory epithelia (Fig 4C and 4D and S5 Movie). These observations were confirmed with two independent double knockdown assays, each of which also included two replicate cultures for each treatment group. Real-time PCR analysis showed 30 nM subthreshold siRNA dose reduced *Dnah6* transcript level by 50–70%, modeling haploinsufficiency associated with heterozygous loss of function (S9 Fig). These findings suggest airway ciliary dysfunction can arise from genetic interaction between *Dnah6* and *Dnai1* or *Dnah5*.

## Discussion

We identified *DNAH6* as a novel ciliome gene that can cause heterotaxy and airway cilia dysfunction similar to PCD. Our initial sequencing analysis of 25 patients identified *DNAH6* mutations in 2 patients with airway ciliary dysfunction, but not in any heterotaxy patients with normal ciliary motion. Functional analysis with gene knockdown in zebrafish embryos and in the human and mouse respiratory epithelia showed *DNAH6* is required for left-right patterning and airway ciliary motion, respectively. Sequencing analysis of an additional 149 heterotaxy patients yielded one patient with a homozygous *DNAH6* mutation, suggesting *DNAH6* also may cause heterotaxy in a recessive manner as in PCD. While a recent study has suggested phenotypes observed with zebrafish morpholino knockdown may reflect off-target effects [15], this is unlikely to account for the phenotypes observed with *dnah6* MO knockdown. First we note the phenotypes observed with MO knockdown included curly tail and left-right patterning defects, both phenotypes robustly associated with disruption of motile cilia function in zebrafish. Second, we independently confirmed with gene knockdown analysis that *Dnah6* is required for motile cilia function not only in zebrafish Kupffer’s vesicle, but also in the human and mouse airway (S3 Table).

*DNAH6* is a putative inner dynein arm component (IDA), and is the ortholog of *Chlamydomonas DHC2* encoding the heavy chain of inner arm species d[16] (component of inner arm group 4). While no mutation in this *Chlamydonomas* heavy chain gene is known, this species is missing in several mutant strains with defects in non-motor subunits. Our EM analysis showed *DNAH6* knockdown caused unexpected central pair defects in human respiratory cilia. The mechanism for this defect remains unknown, but it is worth noting coupling of IDA and central pair defects has been observed in PCD patients with *CCDC39* and *CCDC40* mutations [17, 18]. However, the potential off-target effects of the shRNA cannot be excluded, an inherent limitation of gene knockdown approaches. It is interesting to note thus far no mutation in inner dynein arm (IDA) components has been identified in PCD. This could reflect ascertainment bias, if IDA mutations were to favor heterotaxy over situs inversus or situs solitus, Clinically, a large proportion of human fetuses with heterotaxy die prenatally from complex CHD[2, 19, 20]. Further consideration of this possibility would require examination for heterotaxy/PCD in the human fetal population.

Overall, we identified 8 heteroaxy patients with heterozygous *DNAH6* mutations, 6 of whom also carried heterozygous mutations in *DNHA5*, *DNAI1* and other PCD genes. This enrichment for PCD gene mutations prompted us to experimentally interrogate for potential trans-heterozygous interactions of *DNAH6* with *DNAI1* or *DNAH5*. In zebrafish, heterotaxy was observed upon subthreshold knockdown of *dnah6/dnai1* or *dnah6/dnah5*. Subthreshold knockdown of *Dnah6* in respiratory epithelia from heterozygous *Dnai1* or *Dnah5* mutant mice caused motile cilia defects in the airway epithelia. Together, these studies showed *Dnah6*, a putative IDA component, can genetically interact with two ODA components, *Dnai1* and *Dnah5*.

We note synthetic interactions between inner and outer arm mutations are well known in *Chlamydomonas*. This is associated with immotile cilia and is observed in double mutants lacking the outer arm and inner arm species I1/f, between the outer arm and some monomeric inner arm dyneins, or between dimeric inner arm I1/f dynein and some monomeric inner arm dyneins[21–23]. In combination, these observations suggest functional interdependence between different types of dyneins, further supporting a role for oligogenic interactions functionally linking outer/inner dynein arm components. Interestingly, we note *Chlamydanomonas* ODA/IDA double mutants can exhibit shorter cilia, consistent with the observation of shorter cilia in the respiratory epithelia of heterotaxy patient 9002 harboring the *DNAH6/DNAI1* mutations. We also observed shorter cilia after *Dnah6* knockdown in the human/mouse airway and in zebrafish Kupffer’s vesicle. In contrast, PCD patients with ODA and IDA defects associated with mutations in *DNAAF2* and *DNAAF3* do not exhibit shorter cilia [24, 25]. This suggests *DNAH6* may have a unique role in mediating axonemal doublet stability.

An oligogenic model of disease for heterotaxy is compelling, given the complexity of ciliogenesis. Furthermore, this is consistent with clinical studies showing heterotaxy has largely a complex multi-genic etiology. Our analysis has been focused on assaying the effects of deficiency or haploinsufficiency, since most PCD mutations are in fact loss of function, such as the *DNAI1* mutation found in patient 9002. While analysis of *DNAH6* missense mutations would have been worthwhile, the very large size of the *DNAH6* transcript (12,477bp) precluded the pursuit of such experiments. An oligogenic model of disease is yet to be demonstrated clinically for heterotaxy or PCD, but there are several reports of PCD patients with only a single heterozygous pathogenic PCD mutation, reminiscent of the pathogenic *DNAI1* mutation found in heterotaxy patient 9002 [5, 17, 18, 26]. These findings suggest the possibility that PCD may also arise in an oligogenic context with trans-heterozygous PCD mutations. An oligogenic disease model has been suggested previously for other ciliopathies, such as the triallelic inheritance of *BBS* genes in Bardet-Biedl syndrome and digenic inheritance of *RDS* and *ROM1* loci in retinitis pigmentosa[27, 28]. Additionally, cilia genes *CEP290*, *RPGRIP1L*, *AHI1* and *KIF7* have been reported to act as genetic modifiers in a spectrum of ciliopathies[29–33]. While the latter disorders largely involve nonmotile primary cilia defects, many genes required for primary cilia are also essential for motile cilia function[34]. Finally, we recently observed a high prevalence of cilia related genes among genes recovered causing CHD in a large scale mouse forward genetic screen. These were found in CHD mutant lines with or without heterotaxy, indicating mutations in cilia genes may serve as genetic modifiers in the broader context of CHD pathogenesis [35].

In conclusion, functional analysis of airway ciliary motion in heterotaxy patients identified the novel dynein, *DNAH6*, as a candidate gene for heterotaxy and PCD. Our experimental modeling showed for the first time, *DNAH6* is essential for motile cilia function mediating left-right patterning and in airway mucociliary clearance. Our findings suggest *DNAH6* can act both in a recessive manner and also possibly in trans-heterozygous interactions with other PCD genes to mediate more complex oligogenic model of disease. Overall, these findings have broad relevance for interrogating the complex genetics of heterotaxy, and other ciliopathies. More insights into the genetic etiology of heterotaxy may have clinical translational application in allowing better patient stratification to identify those at risk for post-surgical respiratory complications related to airway ciliary dysfunction. This can help optimize clinical care and improve the prognosis of high-risk heterotaxy/CHD patients.

## Materials and Methods

### Ethics statement

Patient subjects in this study were recruited at Children¹s National Medical Center, Cincinnati Children¹s Hospital, Tokyo Women's Medical University, and Children¹s Hospital of Philadelphia. All of the human subject recruitments were conducted with human study protocols approved by the respective Institutional Review Board (IRB) for the Protection of Human Subjects. Informed consents and assents were obtained at each of the recruitment sites as per approved IRB protocols at the respective institutions. All animal experiments were conducted with approved IACUC protocols at University of Pittsburgh.

### Patient cohorts

25 heterotaxy patients were previously recruited from the Children’s National Medical Center[5]. Additional heterotaxy patients were recruited at Cincinnati Children’s Hospital, Tokyo Women's Medical University, and Children’s Hospital of Philadelphia. Family medical history was obtained and medical records including original imaging studies were reviewed to confirm the cardiac diagnosis. Blood and/or saliva samples were obtained for genetic analysis.

### Animal models

Zebrafish were housed and in the Zebrafish Aquaria facility of University of Pittsburgh. *Dnai1* knockout mice (MGI: 4415703) and *Dnah5* mutant mice (MGI: 3722326) were housed in the mouse facility of the University of Pittsburgh Rango’s Research Center. All animal experiments were conducted with approved IACUC protocols at University of Pittsburgh.

### Exome sequencing analysis for ciliome mutations

Targeted exome sequencing analysis of 25 patients from Children’s National Medical Center was carried out with a custom Agilent SureSelect sequence capture kit (Fig 1A). We complied a comprehensive “ciliome” gene list (S1 Table), which included PCD-causing genes and those present in the Cilia Proteome[36–38]. We also included genes known to regulate left-right patterning, but no mutations were found in any of these genes in our heterotaxy patient cohort. Sequencing was done using ABI SOLiD 4 and Illumina HiSeq2000 sequencer and analyzed using CLCBio Genomic Workbench software. Whole-exome sequencing analysis for 23 heterotaxy patients from Cincinnati Children’s Hospital (Fig 1B) and 6 heterotaxy patients with heterozygous *DNAH6* mutation from Tokyo Women’s Medical University and Children’s Hospital of Philadelphia (Fig 1C, pink) were carried out with NimbleGenSeqCap EZ Human Exome v2.0 capture kit and Illumina HiSeq2000 sequencer. Sequences were analyzed using a GATK pipeline as described. (https://www.broadinstitute.org/gatk/).

Sequence variants were annotated using annovar (www.openbioinformatics.org/annovar) and custom scripts.

Ciliome genes were examined for novel or rare variants, defined as those with <0.8% allele frequency in the 1000 Genomes (www.1000genomes.org) with matched race/ethnicity and NHLBI exome (evs.gs.washington.edu/) databases. Pathogenicity of missense variants were assessed by PolyPhen-2, SIFT and CADD Score algorithms[39–41].

### Ion torrent *DNAH6* amplicon sequencing analysis

PCR amplifications were carried out for all 77 exons and flanking splice junctions for *DNAH6* (primer sequences will be made available upon request). PCR products from each sample were then pooled and sequenced using IonTorrent Personal Genome Machine. Sequence data was analyzed using the CLCBio Genomics Workbench software as described above. The same bioinformatics pipeline as described above for the exome sequencing analysis was used to analyze for *DNAH6* sequence variants. Candidate *DNAH6* coding variants identified were confirmed using Sanger capillary sequencing.

### Mouse embryo in situ hybridization

Partial mouse *Dnah6* cDNA clone obtained from OpenBioSystem (www.openbiosystem.com) was used to generate the *in situ* hybridization probe. *In situ* hybridization analyses of E8.0 mouse embryos were performed using a digoxigenin-labeled *Dnah6* antisense riboprobe following published protocol[42]. After staining to visualize regions of hybridization, the mouse embryos were washed, cleared in 80% glycerol and photographed using a Leica M165FC microscope with ProgRes C14 digital camera.

### Human nasal tissue biopsy and shRNA knockdown in reciliating nasal epithelia

Nasal tissue from healthy human subjects was collected using Rhino-Probe (Arlington Scientific, Springville, UT) curettage of the inferior nasal turbinate and the collected tissue was resuspended in L-15 medium (Invitrogen, CA) for videomicroscopy using a Leica inverted microscope with a 100x oil objective and differential phase contrast optics. The collected human nasal epithelia tissue was cultured using Ultroser G medium (BioSepra, France) on collagen coated plates and grown to confluence[43]. Confluent cultures were treated with lentivirus expressing *DNAH6* short hairpin RNA (sh*DNAH6*) or non-specific scramble control shRNA (sh*SCRAMBLE*) and incubated for 3 hrs at 37°C, after which the cells were removed with collagenase and cells placed in suspension at 37°C on an orbital shaker. By expressing the short hairpin RNA during the process of reciliation, it is possible to assay protein function required for ciliogenesis. Reciliation is usually observed in 7–14 days, and videomicroscopy was carried out at 200 fps using a Phantom v4.2 camera to assess ciliary motion.

### Real time PCR analysis

To assess the efficacy of *DNAH6* knockdown, total RNA from sh*DNAH6* and control sh*SCRAMBLE* treated reciliated human airway epithelial cells were isolated using RNeasy micro kit with on-column DNase I digestion method (QIAGEN). cDNA were prepared and amplified using Ovation RNAseq kit version 2 (NuGen). Real-time PCR was done using Applied Biosystem HT7900 with Power SYBR Green PCR Master Mix (Applied Biosystem) following standard protocol. The ubiquitously expressed *β-actin* gene and cilia-related genes *DNAI1* and *DNAAF2* were used as internal controls. Similar analysis was also conducted using *DNAH6* siRNA treated mouse epithelial cells at different dose (0, 30nM, 50nM), with cilia gene *IFT80* was used as an internal control. Similar experiments were performed to assess the expression of *CCNO*, *MCIDAS and FOXJ1* transcripts in *DNAH6* knockdown and control siRNA treated samples.

### Immunofluorescence analysis of human respiratory cilia

Reciliated respiratory epithelial cells were suspended in cell culture medium and were blast by homogenizer for 3s to break the spheroids. Samples were spread onto glass slides, air-dried and stored at −80°C until use. Cells were treated with 4% paraformaldehyde, 0.1% Triton-X 100 and 1% BSA (all percentages are v/v) before incubation with primary (at least 1 h at room temperature (18–20°C) or overnight at 4°C) and secondary (30 min at room temperature) antibodies. Polyclonal anti-DNALI1, anti-DNAH5 and monoclonal mouse anti-acetylated-α-tubulin and monoclonal mouse gamma–tubulin antibodies were purchased from Sigma. Highly cross-absorbed secondary antibodies (Alexa Fluor 488, Alexa Fluor 546, Alex Fluor 633) were from Molecular Probes (Invitrogen). DNA was stained with DAPI (Sigma). Immunofluorescence images were taken with a Zeiss Apotome Axiovert 200 and processed with Openlab v5.1.

### Transmission electron microscopy of human respiratory cilia

Reciliated human respiratory epithelia were fixed in 2.5% (v/v) glutaraldehyde in 0.1 M sodium cacodylate buffer at 4°C, washed overnight and postfixed in 1% (v/v) osmium tetroxide. After dehydration, the samples were embedded in epoxy resin. Ultrathin sections were obtained from the embedded specimen and the sections were contrasted with Reynold's lead citrate and 4% uranyl acetate in ethanol. Transmission electronmicroscopy was performed with a Philips CM10 and images were captured using a Gatan, Erlangsher CCD camera system.

### Zebrafish gene knockdown with antisense morpholino injections

*dnah6* and *dnai1* antisense morpholinos (MO) were designed and synthesized by GeneTools, LLC (Philomath, OR).

*dnah6* AUG-MO 5’-AAGAATACATGACCAACTTCCCTGC-3’

*dnah6* Spl-MO E24I24 5’- TGAGCGTCATCAGGCCGGACCTGTT-3’

*dnai1*-134914 5’-GGCTGTTTCTCCTCAGACATTTTTC-3’

*dnai1*-80431 5’- TCTTTAAATAACAAGACTGCTCCGC-3’

*dnai1* I8E9 5’- GCTTGAAATCTGAGGTTTGGGTTAA-3’

MO doses ranging from 1-10ng were injected into the 2-cell stage embryos as previously described along with the standard control scrambled MO. Embryos were incubated to the desired stage, then processed for fixation and *in situ* hybridization or direct phenotype visualization under a Leica stereomicroscope and photographed using a Retiga camera (Q-imaging).

### Zebrafish embryo in situ hybridization and immunohistochemistry

Zebrafish embryos were fixed with 4% paraformaldehyde and analyzed by whole mount in situ hybridization using the protocol described previously[44]. The following antisense riboprobes were generated in this study: *spaw[45]*, *myl7[46]* and f*oxa3[47]*. Whole mount immunofluorescence was performed to detect monocilia in Kupffer’s vesicle as described[48]. Acetylated tubulin monoclonal antibody (1:1000; Sigma) and Cy3-conjugated anti mouse IgG antibody (1:500; Jackson ImmunoResearch Laboratories) were used in this study. Images were acquired on a Zeiss LSM700 confocal microscope. Images were processed using ImageJ software (NIH).

### High-speed videomicroscopy of ciliary motion and flow in zebrafish embryo Kupffer’s vesicle

For imaging cilia generated flow in Kupffer's vesicle (KV), zebrafish embryos (6–8 somites) were removed from their chorion and positioned KV facing upwards in a shallow trough cut into a 0.020” thick silicone sheet (AAA Acme Rubber Co, AZ) glued to the bottom of a 35mm glass bottomed culture dish (Willco Wells B.V, Netherlands) and placed under a 63x immersion lens of a upright microscope (Leicia DMLFSA). Microinjection pipettes were fabricated using borosilicate capillary glass (1mm OD/0.75 mm ID) on a P-97 Flaming/Brown micropipette puller (Sutter Instrument Company, CA), and then beveled using a BV-10 microelectrode beveler (Sutter Instrument Company, CA). Micropipettes were filled with 0.20 μm fluorescent polystyrene latex microspheres (Polysciences, PA) and mounted onto a Leicia micromanipulator. A pneumatic PicoPump (World Precision Instruments, Inc., FL) was then used to inject a small amount of fluorescent microspheres into each KV. To track microsphere movement epifluorescence microscopy (excitation 425/60nm, emission 480nm) was conducted using a high-speed CCD camera (Hamamatsu, C9100-12). Video movies of cilia generated flow were captured at a frame rate of 15 frames/sec. The ImageJ software package with MTrackJ plugin was used to manually track fluorescent bead movement within each KV[49]. High-speed (200 fps) movies of KV cilia motility were collected using a Phantom v4.2 camera (Vision Research, NJ).

### siRNA knockdown in reciliating mouse tracheal epithelial

Tracheal tissue from both wildtype and heterozygous *Dnai1* knockout *and Dnah5* mutant mice were collected, diced into very fine pieces and cultured using Ultroser G medium (BioSepra, France) on collagen coated 6-well plates and grown to confluence[43]. Confluent cultures were transfected with different dosage of *Dnah6* siRNA ranging from 0-50nM using Qiagen RNAiMax kit to determine the subthreshold dose. The *Dnah6* siRNA was obtained from Qiagen (SI04413444). Cells were incubated for 4hrs at 37°C, after which the cells were removed with collagenase and placed in suspension at 37°C on an orbital shaker. Reciliation occurs after 7 days in suspension, videomicroscopy was carried out at 200 fps using a Phantom v4.2 camera to assess ciliary motion.

## Supporting Information

S1 Fig *DNAH6* mutations in heterotaxy/PCD patients and parental transmission of the *DNAI1* and *DNAH6* mutations in patient 9002. Sanger sequencing confirmed the father of patient 9002 is heterozygous for the *DNAH6* c.6182G>A allele, but is wildtype for the *DNAI1* allele. In contrast, the mother is heterozygous for the *DNAI1* IVS1+2_3insT allele, but is wildtype for the *DNAH6* allele.
(TIF)

S2 Fig Real time PCR analysis of *DNAH6* transcripts after sh*DNAH6* knockdown. Quantitative analysis by real time PCR showed marked reduction in *DNAH6* transcripts in human respiratory epithelia after sh*DNAH6* knockdown as compared to control. This is confirmed by normalizing using expression of housekeeping gene beta-actin (p-value = 0.00021), as well as cilia-related genes *DNAI1* (p-value = 0.00021) and *DNAAF2* (p-value = 0.00028).
(TIF)

S3 Fig Immunolocalization of DNAH5, DNALI1 and RSPH4A in the ciliary axoneme after *DNAH6* knockdown. Human respiratory epithelia after *DNAH6* knockdown show no apparent change in the distribution of DNAH5 (an outer dynein arm marker), DNALI1 (an inner dynein arm marker) or RSPH4A (central pair component) in the ciliary axoneme. Acetylated tubulin antibody staining (ac-tub; green) was used to visualize the ciliary axoneme. DAPI staining (blue) was used to visualize the nucleus. Note image for DNAH5 staining with DNAH6 knockdown contains two ciliated cells while all the other panels contain only a single multiciliated cell.
(TIF)

S4 Fig Analysis of *CCNO*, *FOXJ1* and *MCIDAS* transcripts with *DNAH6* knockdown. **(A, B)** qPCR analysis showed *CCNO*, *FOXJ1* and *MCIDAS* transcripts were not affected in human and mouse respiratory epithelia after *Dnah6* knockdown as compared to control. **(C)** In situ hybridization showed *foxj1* expression level and pattern did not change in zebrafish embryos after *dnah6* antisense morpholino gene knockdown.
(TIF)

S5 Fig Analysis of heart looping defects with splicing and AUG *dnah6* morpholinos. Both *dnah6* AUG (AUG-MO) and splicing (Spl-MO) morpholinos caused heart looping defects including right sided (R), or straight (St) heart looping phenotypes.
(TIF)

S6 Fig *dnah6 in situ* hybridization analysis of early zebrafish embryos. *In situ* hybridization analysis showed *dnah6* transcripts are ubiquitously expressed as maternally derived transcript in the early zebrafish embryo.
(TIF)

S7 Fig Analysis of splicing and AUG *dnah6* morpholinos. **(A)** RT-PCR analysis confirmed *dnah6* splice morpholino (*dnah6* splMO) disrupted proper splicing of intron 24 in MO-injected embryos vs. control (ContMO). **(B)** The *dnah6* AUGMO provided higher percentage of curly tail phenotype as compared to the *dnah6* splice MO.
(TIF)

S8 Fig Quantification of cilia number in KV. Neither *dnah6* AUG MO or *splice* MO knockdown affects the KV cilia number as compared to the control.
(TIF)

S9 Fig Real time PCR analysis of *Dnah6* transcripts with siRNA knockdown in mouse tracheal epithelial cells. Quantitative analysis by real time PCR showed that subthreshold knockdown with 30nM *Dnah6* siRNA reduced *Dnah6* expression to ~65% compared to the control, 50nM siRNA knockdown further reduced *Dnah6* expression to less than 50%.
(TIF)

S1 Table Ciliome gene list. Table provides HGNC gene symbols and Refseq IDs for genes used for the targeted exome sequencing analysis described in Fig 1A.
(XLSX)

S2 Table *DNAH6* mutations identified in heterotaxy patients. This table provides detailed phenotypic data for heterotaxy patients identified with *DNAH6* mutations, the detailed mutation annotation was also provided in the table.
(PDF)

S3 Table Summary of cilia defects with *Dnah6* knockdown. Summary of the phenotypes observed in zebrafish embryos and in mouse and human respiratory epithelia upon *Dnah6* gene knockdown.
(PDF)

S1 Movie Ciliary motion in reciliated human respiratory epithelia after control shRNA knockdown. Reciliated human respiratory epithelia treated with control shRNA knockdown displayed cilia with normal coordinated ciliary motion and normal cilia waveform. Original movie was captured at 200 fps, movie playback is 30 fps (ie. 15% real time). Scale Bar = 10 μm
(MOV)

S2 Movie Ciliary motion in reciliated human respiratory epithelia after *DNAH6* shRNA knockdown. Reciliated human respiratory epithelia treated with *DHAH6* shRNA displayed cilia with a range of abnormal motility defects, including cilia that exhibited abnormal dyskinetic motion to completely immotile cilia. Original movie was captured at 200 fps, movie playback is 30 fps (ie. 15% real time). Scale Bar = 10 μm
(MOV)

S3 Movie Ciliary motion in zebrafish embryo Kupffer’s vesicle. Kupffer’s vesicle (KV) cilia in control MO injected zebrafish embryos displayed normal rotational movement (top left), but in the *dnah6* MO injected embryos KV cilia displayed a spectrum of motility phenotypes, ranging from more normal motion (top right) to random rotational movement (middle panels) and backward/forward motion (bottom left), to being nearly immotile (bottom right). Original movies were captured at 200 fps, movie playback is 30 fps (ie. 15% real time). Scale Bar = 2.5 μm
(MOV)

S4 Movie Fluid flow in Kupffer’s vesicle as observed with injected beads in zebrafish embryos after antisense MO injection. Microinjection of fluorescent beads into control MO injected zebrafish embryos revealed robust rotational flow, but after *dnah6* MO injections, KV flow (bottom panels) was not observed. Videomicroscopy was conducted using DIC imaging (top left), and epifluorescence microscopy (top right). Scale Bar = 10 μm
(MOV)

S5 Movie Ciliary motion in reciliated mouse airway epithelia from wildtype vs. heterozygous *Dnai1* knockout mice after *Dnah6* siRNA knockdown. In wildtype tracheal epithelial cells, 30nM siRNA knockdown had no effect on ciliogenesis or cilia motility, as the respiratory epithelia continued to show high ciliation density and normal ciliary motion. However, with 50nM knockdown, cilia density was significantly reduced with many areas entirely devoid of cilia, and ciliary motion was abnormal and dyskinetic. In comparison, heterozygous *Dnai1* knockout mouse tracheal epithelia was much more sensitive to the effects of *Dnah6* siRNA knockdown. Knockdown at 30nM *Dnah6*, which had no effect on wildtype tracheal epithelia, caused reduced cilia density and a spectrum of ciliary motion defects ranging from immotile to restricted dyskinetic ciliary motion in heterozygous *Dnai1* knockout mouse tracheal epithelia. These are similar to the phenotypes observed with 50nM *Dnah6* siRNA knockdown in the wildtype tracheal epithelia. This contrasts with the completely immotile cilia observed in the tracheal epithelia of homozygous *Dnai1* knockout mice. Movie was originally captured at 200 fps, and movie playback is set at 30 fps for ease of visualization (ie. 15% real time). Scale Bar = 10 μm
(MOV)

S6 Movie Ciliary motion in reciliated mouse airway epithelia from wildtype vs. heterozygous *Dnah5* mutant mice after *Dnah6* siRNA knockdown. 30nM *Dnah6* siRNA knockdown had no effect on ciliogenesis or cilia motility in wildtype (+/+) tracheal epithelial cells. In comparison, 30nM siRNA in heterozygous Dnah5 (+/m) mutant tracheal epithelia cells showed significantly reduced cilia density, and ciliary motion was largely dyskinetic/immotile. Movie was originally captured at 200 fps, and movie playback is set at 30 fps for ease of visualization (ie. 15% real time). Scale Bar = 10 μm
(MOV)
