# Supplementary figures and images for: DNAH6 and Its Interactions with PCD Genes in Heterotaxy and Primary Ciliary Dyskinesia
Source: PLoS Genet. 2016 Feb 26;12(2):e1005821. doi: 10.1371/journal.pgen.1005821 (PMC4769270; doi:10.1371/journal.pgen.1005821)

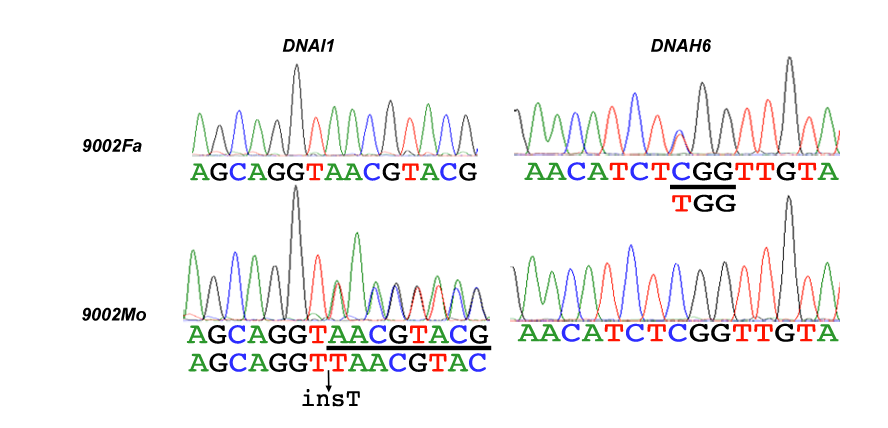

Supplement: S1 Fig — Sanger sequencing confirmed the father of patient 9002 is heterozygous for the DNAH6 c.6182G>A allele, but is wildtype for the DNAI1 allele. In contrast, the mother is heterozygous for the DNAI1 IVS1+2_3insT allele, but is wildtype for the DNAH6 allele. (TIF) [file pgen.1005821.s001.tif]

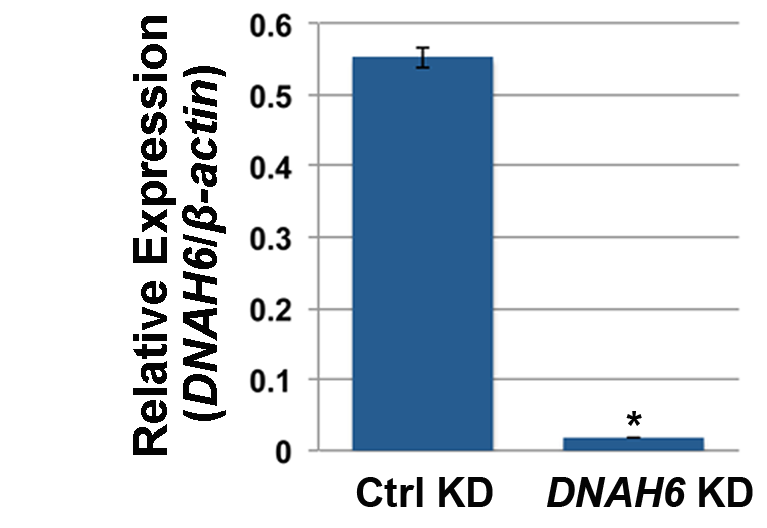

Supplement: S2 Fig — Quantitative analysis by real time PCR showed marked reduction in DNAH6 transcripts in human respiratory epithelia after shDNAH6 knockdown as compared to control. This is confirmed by normalizing using expression of housekeeping gene beta-actin (p-value = 0.00021), as well as cilia-related genes DNAI1 (p-value = 0.00021) and DNAAF2 (p-value = 0.00028). (TIF) [file pgen.1005821.s002.tif]

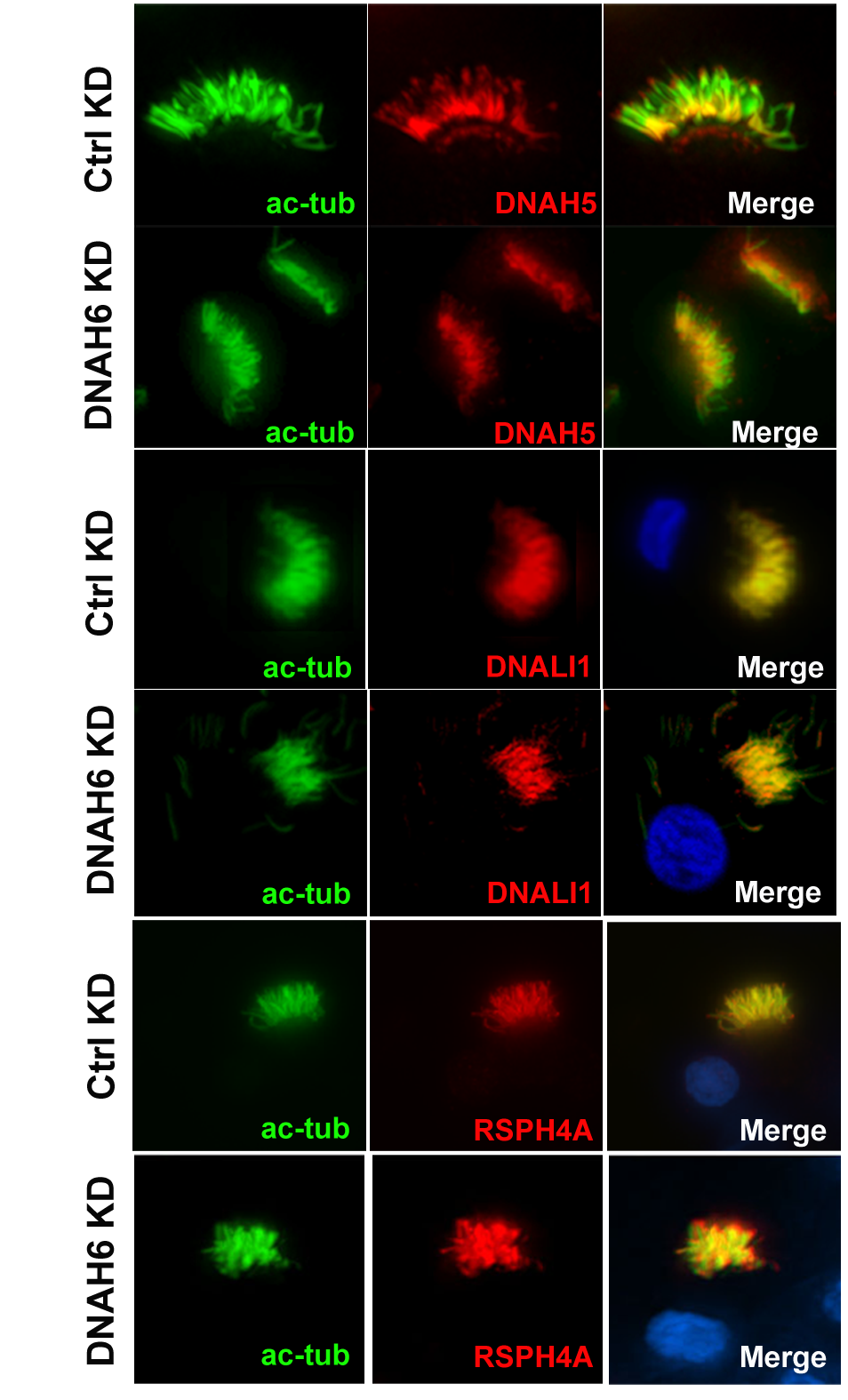

Supplement: S3 Fig — Human respiratory epithelia after DNAH6 knockdown show no apparent change in the distribution of DNAH5 (an outer dynein arm marker), DNALI1 (an inner dynein arm marker) or RSPH4A (central pair component) in the ciliary axoneme. Acetylated tubulin antibody staining (ac-tub; green) was used to visualize the ciliary axoneme. DAPI staining (blue) was used to visualize the nucleus. Note image for DNAH5 staining with DNAH6 knockdown contains two ciliated cells while all the other panels contain only a single multiciliated cell. (TIF) [file pgen.1005821.s003.tif]

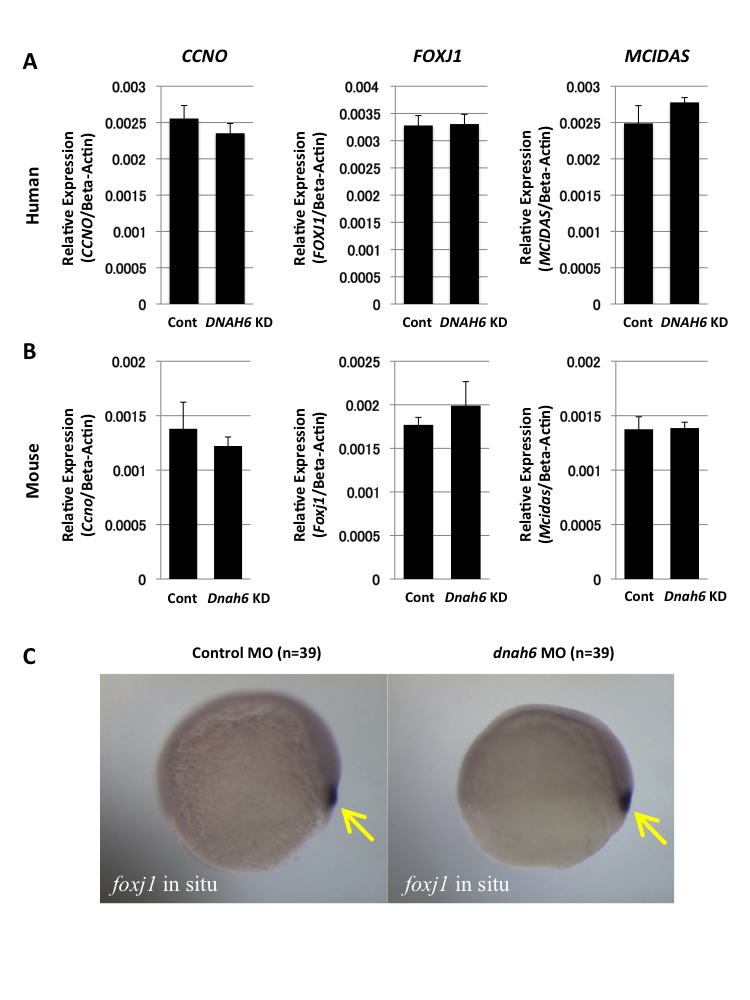

Supplement: S4 Fig — (A, B) qPCR analysis showed CCNO, FOXJ1 and MCIDAS transcripts were not affected in human and mouse respiratory epithelia after Dnah6 knockdown as compared to control. (C) In situ hybridization showed foxj1 expression level and pattern did not change in zebrafish embryos after dnah6 antisense morpholino gene knockdown. (TIF) [file pgen.1005821.s004.tif]

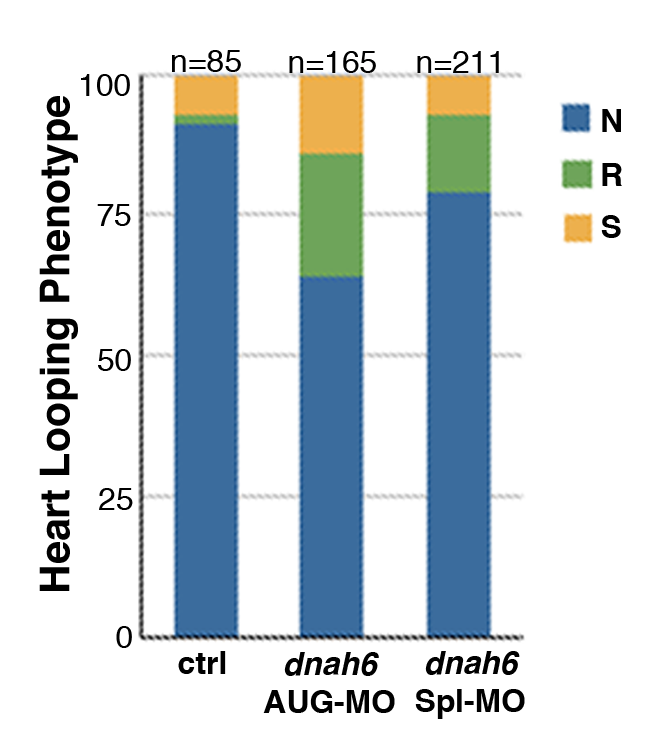

Supplement: S5 Fig — Both dnah6 AUG (AUG-MO) and splicing (Spl-MO) morpholinos caused heart looping defects including right sided (R), or straight (St) heart looping phenotypes. (TIF) [file pgen.1005821.s005.tif]

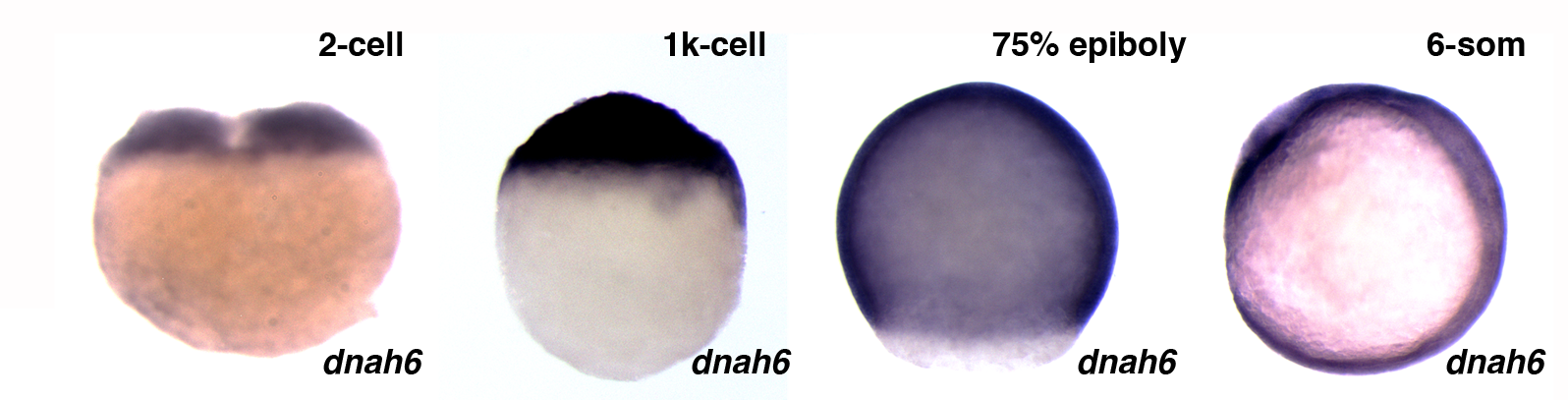

Supplement: S6 Fig — In situ hybridization analysis showed dnah6 transcripts are ubiquitously expressed as maternally derived transcript in the early zebrafish embryo. (TIF) [file pgen.1005821.s006.tif]

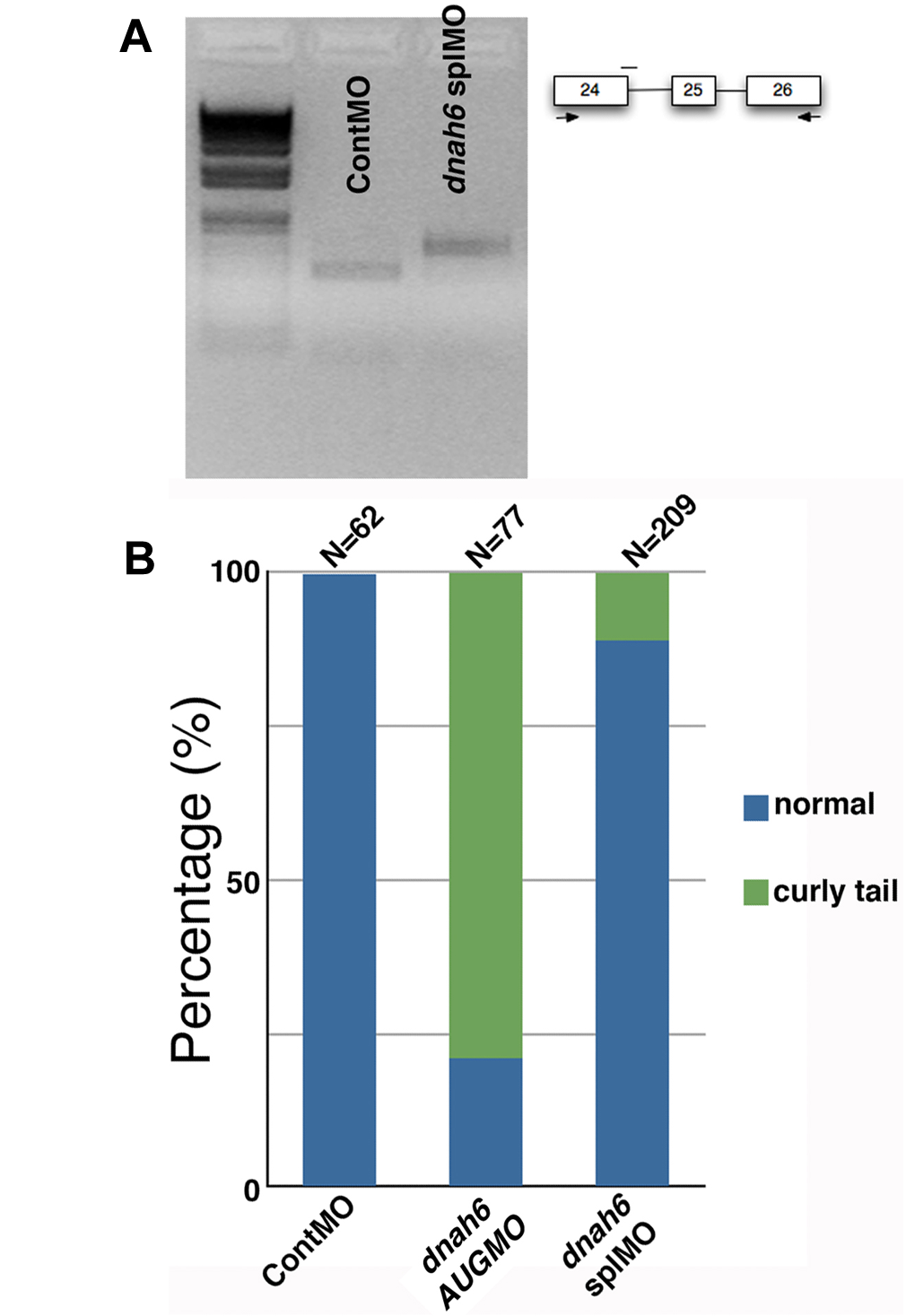

Supplement: S7 Fig — (A) RT-PCR analysis confirmed dnah6 splice morpholino (dnah6 splMO) disrupted proper splicing of intron 24 in MO-injected embryos vs. control (ContMO). (B) The dnah6 AUGMO provided higher percentage of curly tail phenotype as compared to the dnah6 splice MO. (TIF) [file pgen.1005821.s007.tif]

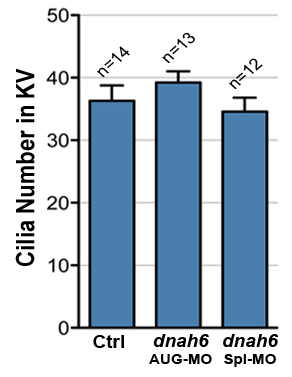

Supplement: S8 Fig — Neither dnah6 AUG MO or splice MO knockdown affects the KV cilia number as compared to the control. (TIF) [file pgen.1005821.s008.tif]

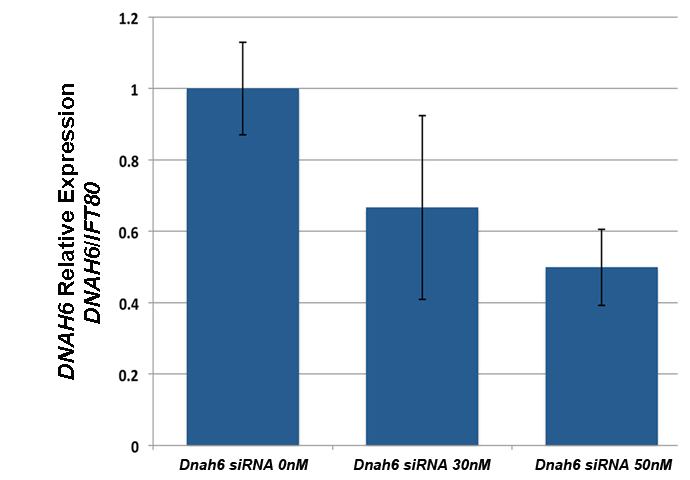

Supplement: S9 Fig — Quantitative analysis by real time PCR showed that subthreshold knockdown with 30nM Dnah6 siRNA reduced Dnah6 expression to ~65% compared to the control, 50nM siRNA knockdown further reduced Dnah6 expression to less than 50%. (TIF) [file pgen.1005821.s009.tif]
