# Supplementary material for: DNAH6 and Its Interactions with PCD Genes in Heterotaxy and Primary Ciliary Dyskinesia
Source: PLoS Genet. 2016 Feb 26;12(2):e1005821. doi: 10.1371/journal.pgen.1005821 (PMC4769270; doi:10.1371/journal.pgen.1005821)
Supplement: S2 Table — This table provides detailed phenotypic data for heterotaxy patients identified with DNAH6 mutations, the detailed mutation annotation was also provided in the table. (PDF) [file pgen.1005821.s011.pdf]

Table S2 *DNAH6* mutations identified in heterotaxy patients.

Patient	Race/ Ethnicity	Sex	Cardiac and Noncadiac Laterality Defect¥	Cilia Function†	Gene	Nucleotide Change	Protein Change	Zygosity	Allele Frequency*
**Children's National Medical Center**									
9002	White	M	asplenia, midline liver, malrotation, dextrogastria, interrupted IVC with azygous continuation, RAA, LSVC to coronary sinus	*CD*	*DNAH6*	c.6182G>A	p.R2061Q	het	Novel
9027	Hispanic	F	left atrial isomerism, left bronchial isomerism, TOF, LSVC drains into L atrium, L atrial isomerism, RAA, hypoplastic PAs with collaterals	*CD*	*DNAH6*	c.4451A>G	p.D1484G††	het	Novel
**Cincinnati Children's Hospital**									
T17	White	M	D-TGA, VSD, pulmonary stenosis	*n.d*	*DNAH6*	c.C9874T	p.R3292C ††	homo	Novel
**Tokyo Women's Medical University**									
JP2090	Asian	F	asplenia, DORV, AVSD, PA	*n.d.*	*DNAH6*	c.G11566A	p.E3856K††	het	Novel
JP2637	Asian	F	polysplenia, CA, SV, PS	*n.d.*	*DNAH6*	c.G612A	p.M204I††	het	Novel
JP3617	Asian	F	asplenia, SLV	*n.d.*	*DNAH6*	c.G9097A	p.D3033N	het	Novel
JP3634	Asian	M	asplenia, SRV	*n.d.*	*DNAH6*	c.C1820G	p.A607G††	het	Novel
**Children's Hospital of Philadelphia**									
GOLD53	White	F	dextrocardia, CA, SV, interrupted IVC with azygous continuation to the right SVC, RAA, polysplenia, midline liver, possible malrotation; scoliosis	*n.d.*	*DNAH6*	c.T2369C	p.I790T	het	0.06%
GOLD54	Black	M	midline liver, right-sided stomach/pancreas, malrotation, apslenia, trilobed lungs bilaterally, CAVC, CA, SV, aortic valve atresia, hypoplastic aortic arch, TAPVR, interrupted IVC	*n.d.*	*DNAH6*	c.T8509G	p.F2837V††	het	0.01%

¥ Phenotype abbreviations**:**
AVSD: atrioventricular septal defect; CA: common atrium; CAVC: common atrioventricular canal; DORV: double outlet right ventricle; IVC: Inferior vena cava; LV: left ventricle; RAA: right aortic arch; SV: single ventricle; SVC: superior vena cava; TAPVR: total anomalous pulmonary venous return; TGA: transposition of the great arteries; TOF: tetralogy of Fallot; VSD: ventricular septal defect.

* Allele frequencies were derived from NHLBI exome database. (http://evs.gs.washington.edu/EVS/)

† CD: airway ciliary dysfunction as determined by the finding of low nasal nitric oxide and abnormal airway ciliary motion observed by videomicroscopy.

†† Missense *DNAH6* mutations predicted to be deleterious by PolyPhen-2, SIFT and CADD Score[35-37].
