# Supplementary material for: DNAH6 and Its Interactions with PCD Genes in Heterotaxy and Primary Ciliary Dyskinesia
Source: PLoS Genet. 2016 Feb 26;12(2):e1005821. doi: 10.1371/journal.pgen.1005821 (PMC4769270; doi:10.1371/journal.pgen.1005821)
Supplement: S3 Table — Summary of the phenotypes observed in zebrafish embryos and in mouse and human respiratory epithelia upon Dnah6 gene knockdown. (PDF) [file pgen.1005821.s012.pdf]

Table S3 Summary of cilia defects with *DNAH6* knockdown

Tissue	Cilia Defects Observed with *DNAH6* Knockdown
Zebrafish Kuppfer's Vesicle	shorter cilia, largely immotile/dyskinetic
Human Nasal Epithelia	sparse cilia, cilia of smaller caliper mostly immotile/dyskinetic,
Mouse Tracheal Epithelia	sparse ciliation, most areas bald, immotile/dyskinetic ciliary motion
